# Pneumothoraxes after CT-guided percutaneous transthoracic needle aspiration biopsy of the lung: A single-center experience with 3426 patients

**DOI:** 10.5578/tt.20239909

**Published:** 2023-03-10

**Authors:** G. POLAT, Ö. ÖZDEMİR, D.S. UNAT, G. KARADENİZ, A. AYRANCI, Ö.S. UNAT, M. BÜYÜKŞİRİN, A. MAVİŞ, S. YAZGAN

**Affiliations:** 1 Department of Thoracic Diseases, Health Sciences University Faculty of Medicine, İzmir, Türkiye; 2 Clinic of Thoracic Diseases, Dr Suat Seren Chest Diseases and Surgery Training and Research Hospital, University of Health Sciences, İzmir, Türkiye; 3 Clinic of Thoracic Diseases, İzmir Kemalpaşa State Hospital, İzmir, Türkiye; 4 Clinic of Thoracic Diseases, Çiğli Training and Research Hospital, University of İzmir Bakırçay, İzmir, Türkiye; 5 Department of Thoracic Diseases, Ege University Faculty of Medicine, İzmir, Türkiye; 6 Clinic of Radiology, Dr. Suat Seren Chest Diseases and Surgery Training and Research Hospital, University of Health Sciences, İzmir, Türkiye; 7 Clinic of Thoracic Surgery, Dr. Suat Seren Chest Diseases and Surgery Training and Research Hospital, University of Health Sciences, İzmir, Türkiye

**Keywords:** Computed tomography, percutaneous transthoracic needle aspiration biopsy, pneumothorax

## Abstract

**ABSTRACT:**

Pneumothoraxes after CT-guided percutaneous transthoracic needle aspiration biopsy of the lung: A single-center experience with 3426 patients

**Introduction:**

The purpose of this study is to determine how long patients who developed pneumothorax were followed up on in the emergency department, how many patients required chest tube placement, and what factors influenced the need for a chest tube in patients who underwent computed tomography (CT)-guided percutaneous transthoracic fine needle aspiration biopsy (PTFNAB).

**Materials and Methods:**

Patients who developed pneumothorax following CT-guided PTFNAB were analyzed retrospectively. In cases with pneumothorax, the relationship between chest tube placement and the size of the lesion, the lesion depth from the pleural surface, the presence of emphysema, and the needle entry angle were investigated. It was determined how long the patients were followed up in the emergency department, when a chest tube was placed, and when patients who did not require chest tube placement were discharged.

**Results:**

CT-guided PTFNAB was performed in 3426 patients within two years. Pneumothorax developed in 314 (9%) cases and a chest tube was placed in 117 (37%). The risk factor for chest tube placement was found to be the lesion depth from the pleural surface. The lesion depth from the pleural surface of >24 mm increased the risk of chest tube placement by 4.8 times. Chest tubes were placed at an average of five hours (5.04 ± 5.57).

**Conclusion:**

This study has shown that in cases with pneumothorax that required chest tube placement, the lesion depth from the pleural surface is a risk factor. Patients who developed pneumothorax on CT during the procedure had chest tubes placed after an average of five hours.

## Abstract (tr)

**ÖZ:**

Akciğere BT kılavuzluğunda perkütan transtorasik iğne aspirasyon biyopsisi sonrası pnömotorakslar: 3426 hasta ile tek merkez deneyimi

**Giriş:**

Bu çalışmanın amacı, bilgisayarlı tomografi rehberliğinde perkütan transtorasik ince iğne aspirasyonu (TTİAB) uygulanan ve buna bağlı olarak pnömotoraks gelişen hastaların kaç tanesine transtorasik göğüs tüpü uygulaması gerektiği, hangi faktörlerin göğüs tüpü ihtiyacını etkilediği ve bu hastaların ne kadar süre acilde takip edildiğini değerlendirmektir.

**Materyal ve Metod:**

BT kılavuzluğunda TTİAB sonrası pnömotoraks gelişen hastalar geriye dönük olarak incelendi. Pnömotorakslı olgularda göğüs tüpü uygulamasıyla lezyonun boyutu, lezyonun plevral yüzeyden derinliği, amfizem varlığı ve iğne giriş açısı arasındaki ilişki araştırıldı. Hastaların acil serviste ne kadar süre takip edildiği ne zaman göğüs tüpü takıldığı ve göğüs tüpü takılmasına gerek olmayan hastaların ne zaman taburcu edildiği değerlendirildi.

**Bulgular:**

İki yıl içinde 3426 hastaya BT kılavuzluğunda TTİAB uygulandı. Üç yüz on dört (%9) olguda pnömotoraks gelişti ve 314 olgunun 117’sine (%37) göğüs tüpü takıldı. Lezyonun plevra yüzeyinden derinliği göğüs tüpü ihtiyacı için risk faktörü olarak bulundu. Plevral yüzeyden uzaklığı 24 mm’den fazla olan lezyon derinliği göğüs tüpü gerekliliği riskini 4,8 kat artırdı. Hastalara ortalama beş saatte (5,04 ± 5,57) göğüs tüpü yerleştirildi.

**Sonuç:**

Bu çalışma, TTİAB yapılıp pnömotoraks olan olgularda göğüs tüpü ihtiyacı için lezyonun plevral yüzeyden derinliğinin bir risk faktörü olduğunu ortaya koydu. Pnömotoraks tanısı konulan hastaların ortalama takibi beş saat olduğu görüldü

## Introduction

Pathological diagnosis of pulmonary lesions is important for precise treatment planning. Many diagnostic methods are available, including computed tomography (CT) guided biopsy, bronchoscopyguided transbronchial biopsy, and surgical excision of the lesion. However, the process must be quick, practical, and cost-effective.

Computed tomography guided percutaneous transthoracic needle aspiration biopsy (PTFNAB) is a nonsurgical invasive diagnostic procedure that is a viable alternative to surgical biopsy. As with all invasive procedures, it may result in complications; therefore, prior familiarity with the method is essential, and it should not be conducted on high-risk patients ( 1 ). The diagnostic accuracy of CT-guided PTFNAB varies between 82-98% ( 2 ). Pneumothorax is the most common complication of the procedure ( 3 , 4 ). Chest tube placement is required in 5-36.1 percent of cases where pneumothorax develops as a result of CT-guided PTFNAB, which increases hospitalizations and costs ( 5 , 6 ).

Percutaneous pulmonary interventions are wellknown procedures. In 1828, the first paper on the subject was published ( 1 ). It was not initially preferred due to the high rate of hemoptysis and pneumothorax. Multiple pleural punctures and the use of thick needles increase the risk of pneumothorax, but the diagnostic yield depends on the number of samples obtained ( 7 ). Two important points to consider when choosing a diagnostic procedure are complication rate and diagnostic performance. After the procedures were started to be performed under CT guidance, complication rates decreased, and diagnostic efficiency increased.

Thanks to the technical advances in CT imaging, unexplained pulmonary lesions are diagnosed with an increasingly lower complication rate. To develop an effective treatment strategy, it is critical to distinguish between benign and malignant tumors, as well as primary lung tumors and metastases. Computed tomography guided biopsy is a method that helps achieve these objectives. Computed tomography guided biopsy method is more appropriate for peripheral lesions. However, it is now also used for central lesions.

This study aims to retrospectively analyze how long patients who developed pneumothorax were followed up in the emergency department, how many patients required chest tube placement, and what factors influenced the need for a chest tube in patients who underwent CT-guided PTFNAB.

## MATERIALS and METHODS

The study was conducted according to good clinical practices and the Declaration of Helsinki. This retrospective study was approved by the ethics committee of Dr. Suat Seren Chest Diseases and Surgery Education and Training Hospital, İzmir (Decision no: 28, Date: 23.11.2020).

Patients who developed pneumothorax after CT-guided PTFNAB and were referred to the emergency room for follow-up by the radiologist were studied retrospectively.

Computed tomography scans were performed on patients prior to the procedure using a 16-slice helical CT device. Scanning parameters were 120 kV, 220- 240 mA, and 1.25 to 2 mm section thickness. Continuous images were reconstructed with 1-1.25 mm thickness. The clinical decision to perform a percutaneous CT-guided PTFNAB was determined by consensus among pulmonologists, interventional radiologists, and thoracic surgeons based on the location of the lesion and the patient’s underlying medical history. In the pre-procedure examination, the most suitable access method was determined. The needle entry angle and the distance between the skin and pleura were measured. Fine-needle aspiration was performed with a 22-gauge Chiba aspiration needle according to CIRSE criteria (8). The choice of the biopsy device(s) and the number of samples obtained was based on the cytopathologist’s preliminary evaluation and the presence of an acute complication that necessitated the termination of the procedure. If a specimen was considered inadequate by the cytopathologist, all attempts to provide an adequate specimen were made, including additional biopsies, obtaining biopsies from another quadrant of the nodule, and/or needle repositioning. Patients who developed pneumothorax detected by CT during the procedure were referred to the emergency department. A chest tube was inserted when a displaced lateral visceral pleural line was visible on chest radiographs or if the patient was symptomatic even though the pneumothorax was small.

In addition to demographic characteristics, it was determined how long the patients were followed up in the emergency department, when a chest tube was placed, and when the patients who did not need a chest tube placement were discharged. In cases with pneumothorax, the relationship between the development of pneumothorax and the age of the patients, the size of the lesion, the lesion depth from the pleural surface, the presence of emphysema in the same lobe, and the needle entry angle were investigated.

### Statistical Analysis

The data obtained in the study were entered into the SPSS (18.0) software. The compatibility of continuous variables to normal distribution was investigated. The Student’s t-test was used to compare independent subgroups of appropriate variables, and mean and standard deviation data were given. The MannWhitney U test was used to evaluate independent subgroups of variables for those that did not have a normal distribution. The most appropriate cut-off value was determined according to the Youden index by performing ROC analysis in independent variables. Sensitivity and specificity were calculated using different cut-off values. The odds ratio (OR) was calculated using the Backward-Wald method.

Cross-squares were created, and their distributions were made using the Chi-square test method. In all these comparisons, variables with p values below 0.2 were taken into Cox regression analysis, and OR was calculated by performing multivariate analysis. All comparison tests and Type 1 error coefficient were determined as alpha 0.05 and were tested in a twotailed test.

## RESULTS

**Figure 1:**
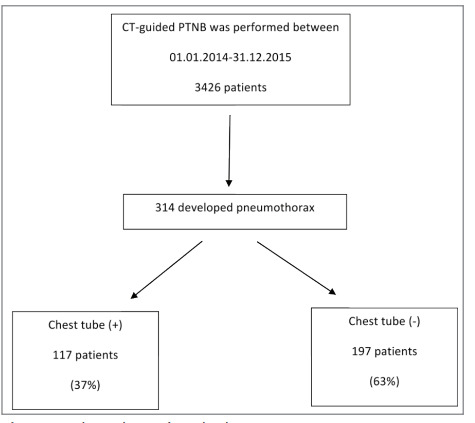
Flow chart of study design.

Computed tomography guided PTFNAB was performed in 3426 patients within two years. It was determined that 314 (9%) of these cases developed pneumothorax during the procedure and they were referred to the emergency department (Figure 1). Of the 314 patients included in the study, 271 (86.3%) were male, 43 (13.7%) were female, and the mean age was 63.78 ± 9.99 (Table 1).

A chest tube was placed in 117 (37%) of 314 patients. Chest tube placement rates were 39.1% (106/271) in men and 25.6% (11/43) in women (p= 0.13). No significant difference was found between the mean age of the patients with and without chest tube placement (p= 0.43) (Table 2).

A chest tube was placed in 79 (39.5%) of 200 patients with emphysema in the same lobe and 38 (33.8%) of those without emphysema. There was no statistically significant difference between these groups (p= 0.28).

The mean size of the lesions was 34.67 ± 18.73 mm and the lesion depth from the pleural surface was 27.21 ± 19.41 mm (Table 1). The mean lesion size in patients with and without chest tubes was 37.14 ± 19.97 mm and 33.21 ± 17.85 mm, respectively (p= 0.09) (Table 2).

The lesion depth from the pleural surface was 34.62 ± 19.80 mm in patients with chest tube placement, while it was 22.81 ± 17.82 mm in patients without chest tube placement (p< 0.01) (Table 2). While there was no relationship between the size of the lesion and chest tube placement, the lesion depth from the pleural surface was significantly different between the two groups. ROC analysis was performed to estimate the chest tube requirement according to the lesion depth from the pleural surface. If the lesion depth from the pleural surface was >24 mm, the sensitivity was 70.1% and the specificity was 64.5% (Figure 2,3).

All these variables were included in the multivariate logistic regression analysis. The OR was calculated according to the Backward-Wald method. The OR was calculated as 4.80 [95% Confidence Interval (CI) 2.88-8.01] for the lesion depth from the pleural surface >24 mm, and 1.82 (95% CI 1.07-3.09) for the presence of emphysema in the same lobe (Table 3).

PTFNAB was performed in 59 patients with an angle of 30-45° and in 255 patients with an angle of 75-90°. There was no significant relationship between needle entry angle and chest tube placement (p= 0.99) (Table 2).

While the tube was placed in patients who required a chest tube placement after an average of five hours, the patients who did not need a chest tube placement were followed up for an average of 7.2 hours. A statistically significant difference was found between the two groups (p< 0.01) (Table 2).

**Table 1:** Patient demographics and lesion characteristics

Variable	Total (n= 314) n (%)
Gender	
Male	271 (86.3)
Female	43 (13.7)
Emphysema	
Present	200 (63.7)
Absent	114 (36.3)
Needle angle	
30-45 ^°^	59 (18.8)
75-90^°^	255 (81.2)
Follow-up time in emergency (hour) (mean ± SD)	6.41 ± 5.57
Time to thoracostomy (hour) (mean ± SD)	6.33 ± 9.16
Lesion depth from the pleural surface (mean ± SD)	27.21 ± 19.41

SD: Standard deviation.

**Table 2:** Comparison of the characteristics of cases with and without chest tube placement

Variable	Tube thoracostomy (+) (n= 117) n (%)	Tube thoracostomy (-) (n= 197) n (%)	p
Variable	Total (n= 314) n (%)
Age (mean ± SD)	63.09 ± 10.27	64.19 ± 9.83	0.43
Gender			
Male	106 (90.6)	165 (83.8)	0.13
Female	11 (9.4)	32 (16.2)	
Emphysema			
Present	79 (67.5)	121 (61.4)	0.28
Absent	38 (32.5)	76 (38.6)	
Needle angle			
30-45^°^	22 (18.8)	37 (18.8)	0.99
75-90^°^	95 (81.2)	160 (81.2)	
Follow-up time in emergency (hour) (mean ± SD)	5.04 ± 5.57	7.22 ± 5.43	<0.001
Time to thoracostomy (hour) (mean ± SD)	N/A	N/A	N/A
Size of the lesion (mm) (mean ± SD)	37.14 ± 19.97	33.21 ± 17.85	0.089
Lesion depth from the pleural surface (mean ± SD)	34.62 ± 19.80	22.81 ± 17.82	<0.001

SD: Standard deviation.

**Figure 2:**
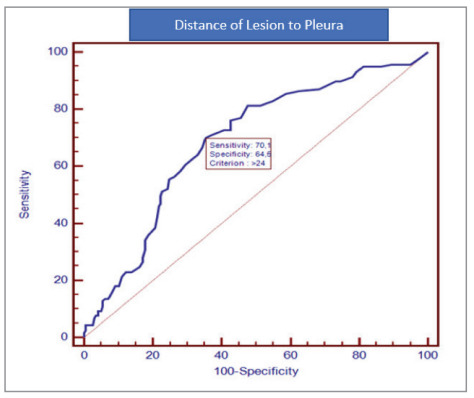
ROC analysis according to the cut-off value of the lesion depth from the pleural surface.

**Figure 2:**
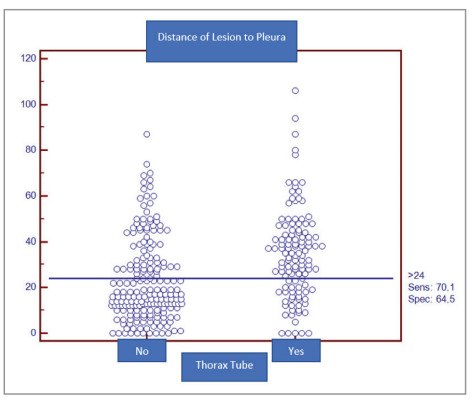
Dot diagram for the lesion depth from the pleural surface.

**Table 3:** Results of the multivariate logistic regression analysis predicting pneumothorax

	Odds ratio	95% confidence interval	p
Lesion depth from the pleural surface	4.80	2.88-8.01	<0.001
Presence of emphysema	1.82	1.07-3.09	0.026

## DISCUSSION

In our study, it was observed that pneumothorax developed in 9% of the patients who underwent CT-guided PTFNAB. A chest tube was placed in 37% of those who developed pneumothorax and in 3% when all cases with PTFNAB were taken into consideration. It was determined that the most important risk factor for chest tube requirement in cases with pneumothorax was the lesion depth from the pleural surface. The lesion depth from the pleural surface of >24 mm increased the risk of chest tube placement 4.8 times, while the presence of emphysema in the same lobe increased by 1.8 times. It was observed that the chest tube was placed in the patients at the fifth hour on average.

While the rate of pneumothorax development after CT-guided PTFNAB varied between 15-54% in different studies, it was 9% in our study, which was lower than the literature (9-11). This lower complication rate may arise from the differences between previous studies and the current study regarding procedural or lesion characteristics such as the diameter of the needle, entry angle, or lesion depth. It was observed that the chest tube was placed in 3% of all cases, which is consistent with previous publications which reported chest tube placement at 1.4-16.7% (9,10). However, chest tubes were placed in 37% of pneumothorax patients, which was greater than in other publications, which reported that chest tubes were placed in 5-36.1% of pneumothorax cases (5,6).

There was no significant difference in age or gender between the groups with and without chest tube placement in our study. While there was no difference in terms of gender in other studies, there are studies showing that the rate of pneumothorax requiring chest tube placement may increase with age. It has been reported that especially elderly patients may have a greater sense of dyspnea due to their lung reserves and that the drainage rate may increase. It has also been reported that the drainage after pneumothorax is higher, as elderly patients may fail to lie on the biopsy side due to pain and slowed mobility ( 12 ).

We investigated the effect of the needle angle on the chest tube requirement since there are studies about the increased risk of pneumothorax associated with the needle angle ( 13 ). There was no statistically significant relationship between needle entry angle and chest tube requirement.

When the cases with and without chest tube placement were compared in terms of the presence of emphysema in the same lobe, there was no statistically significant difference between the groups. In studies investigating risk factors for pneumothorax, the presence of emphysema is an important risk factor ( 14 , 15 ). In addition, although it has been shown in studies that it is an important risk factor in terms of chest tube placement in cases with pneumothorax, it was not found as a risk factor in our study as compared with the chi-square test. However, gender, lesion size, lesion depth from the pleural surface, and the presence of emphysema were included in multivariate logistic regression analysis. In the analysis, one of the two independent variables was emphysema according to the backward-Wald method. OR was found to be 1.8 for emphysema. Patients with emphysema can quickly become dyspneic due to limited respiratory reserve. Therefore, it is important to place the chest tube quickly ( 9 , 16 , 17 , 18 ).

Studies indicate that the size of the lesion affects the risk of pneumothorax. Since it is difficult to obtain sufficient material in smaller lesions, more punctures are required, and the risk of pneumothorax increases. Therefore, as the size of the lesion decreases, the risk of developing pneumothorax increases ( 14 , 19 ). However, studies have not reported lesion size as a risk factor for tube placement. In our study, we found that the size of the lesion was not a risk factor for tube drainage.

In our study, the lesion depth from the pleural surface was found to be an important risk factor for chest tube placement. It is known that the increased length of the lung parenchyma crossed by the needle is a risk factor for pneumothorax. It was suggested that a chest tube was required, as the increased length of the lung parenchyma crossed by the needle resulted in more pneumothorax. Similar to our study, Hiraki et al. found that the lesion depth from the pleural surface is an independent risk factor for tube drainage ( 10 ).

The development of iatrogenic pneumothorax is critical when evaluating the importance of CT-guided biopsy and causes the method to be declined. It is considerably worse to develop pneumothorax severe enough to have a chest tube. To limit the risk of pneumothorax, fine needles are employed.

Furthermore, studies demonstrate that the risk of pneumothorax varies with position. The aim of these studies is to identify the position where the pneumothorax develops the least and to limit the risk. One study found that positioning the patient with the biopsy side down lowers the occurrence of pneumothorax. In this study, the effect of position on chest tube placement was also investigated and it was observed that the side-down position also reduced the chest tube placement ( 14 ). But we ensured that the position of the patient is such that the lesion could be reached most easily. Therefore, the effect of position on chest tube placement could not be evaluated.

In a study conducted, it was reported that rapid removal of the needle and placing the patient on the biopsy side decreased pneumothorax development requiring drainage in PTFNAB ( 20 ). This could not be evaluated in our study.

The most important limitation of the study is that it is a single-center retrospective study and therefore there is a lack of data. More risk factors could be investigated for chest tube placement, but the number of risk factors we could evaluate was limited due to the lack of data.

Our study is one of the few studies evaluating risk factors in terms of chest tube placement. Studies are generally planned to evaluate risk factors for pneumothorax.

## CONCLUSION

In conclusion, the lesion depth from the pleural surface was found to be a risk factor in patients with pneumothorax and chest tube placement. In cases where the lesion depth is more than 24 mm, it should be considered that the rate of chest tube placement increases four times and care should be taken in these cases. In cases that underwent chest tube placement, pneumothorax reached the size that required a chest tube at the fifth hour. Therefore, it was concluded that patients with pneumothorax, which were monitored on CT during the procedure but not noticed on chest radiography, should be followed up in the emergency department.

### Acknowledgments

There was no financial support received during the writing of this article. Patients who participated in this study provided informed consent. The authors who contributed to the article had no conflict of interest.

### Ethical Committee Approval:

This study was approved by İzmir Dr. Suat Seren Chest Diseases and Surgery Education and Training Hospital, Ethics Committee (Decision no: 31, Date: 29.12.2020).

### Conflict of Interest

The authors declare that they have no conflict of interest.

## AUTHORSHIP CONTRIBUTIONS

Concept/Design: GP, MB, ÖÖ

Analysis/Interpretation: GK, AA, SY, AM

Data acqusition: DSU, ASU, ÖÖ

Writing: GP, AA, MB, SY

Clinical Revision: OSU, DSU, AM

Final Approval: MB, GP, GK
